# Application of Dendrimers for the Treatment of Infectious Diseases

**DOI:** 10.3390/molecules23092205

**Published:** 2018-08-31

**Authors:** Zandile Mhlwatika, Blessing Atim Aderibigbe

**Affiliations:** Department of Chemistry, University of Fort Hare, Alice Campus, Eastern Cape 5700, South Africa; 201103519@ufh.ac.za

**Keywords:** dendrimers, polymers, antivirals, parasites, drug delivery

## Abstract

Dendrimers are drug delivery systems that are characterized by a three-dimensional, star-shaped, branched macromolecular network. They possess ideal properties such as low polydispersity index, biocompatibility and good water solubility. They are made up of the interior and the exterior layers. The exterior layer consists of functional groups that are useful for conjugation of drugs and targeting moieties. The interior layer exhibits improved drug encapsulation efficiency, reduced drug toxicity, and controlled release mechanisms. These unique properties make them useful for drug delivery. Dendrimers have attracted considerable attention as drug delivery system for the treatment of infectious diseases. The treatment of infectious diseases is hampered severely by drug resistance. Several properties of dendrimers such as their ability to overcome drug resistance, toxicity and control the release mechanism of the encapsulated drugs make them ideal systems for the treatment of infectious disease. The aim of this review is to discuss the potentials of dendrimers for the treatment of viral and parasitic infections.

## 1. Introduction

Infectious diseases are caused by microorganisms such as bacteria, viruses, parasites or fungi [1]. The diseases can be transmitted by bites from insects or animals; or they can be spread directly or indirectly from one person to another or through contaminated food, plants, soil or water [2]. People with compromised immune systems and children are the most affected by the diseases [3,4]. Infectious diseases can be treated but their treatment is hindered by simultaneous resistance to multiple drugs [3,5]. Due to the development of drug resistance by infectious agents, several researchers have developed drug delivery systems for the treatment of infectious diseases.

Viruses are microorganisms living cells that replicate only within living cells by using the enzyme systems of the cells. Viral infections in human include herpes, influenza, HIV/AIDS etc. [6]. About 7.7% of deaths in South Africa were caused by influenza and pneumonia in 2011 [7]. A study conducted in South Africa also showed that about 44% of HIV patients were likely to have influenza co-infection [8]. AIDS still remains the top 10 leading causes of death in South Africa [9]. Although the number of infectious diseases is still high globally, the overall death rate is decreasing. This may be due to the improved service delivery, improved access to healthcare centre, good nutrition, and better education about infectious diseases [10]. However, in 2010, the number of death caused by infectious diseases had decreased [11]. The World Health Organization (WHO) reported that there is a possibility of a million deaths due to infectious diseases by 2050 indicating that there is a pressing need to develop therapeutics that can treat infectious diseases effectively [12].

Drug delivery systems are potential therapeutic carriers which offer several advantages when compared to the conventional drugs used for the treatment of infectious diseases. Some examples of delivery systems used for the treatment of infectious diseases are polymer-drug conjugates, micelles, nanogels, hydrogel, emulsion, dendrimers etc. The unique properties of dendrimers as drug delivery systems make them potential devices for the treatment of infectious diseases [13]. Some of the advantages are: reduced toxicity, increased specificity which results in the protection of the healthy cell, tissues and organs from the toxic side effects of the drug; improved bioavailability; extended half-life resulting in reduced kidney clearance and protection of the incorporated drugs from premature degradation by enzymatic reactions and other scavenging mechanisms. This review will report the biological efficacy of dendrimers in the treatment of infectious diseases.

## 2. Parasitic Infections

A parasite is an organism that lives within or on a host, and its survival is dependent on the host. Some parasitic diseases are easily treated, while some are not. Common parasitic diseases are malaria, leishmaniasis, schistosomiasis and toxoplasmosis [14,15].

### 2.1. Malaria

Malaria is a parasitic disease caused by the genus *Plasmodium* parasite carried by female *Anopheles* mosquitoes [16]. There are five types of *Plasmodium* parasite that infect humans, including: *P. ovale*, *P. malariae*, *P. knowlesi*, *P. vivax* and *P. falciparum* [17]. *Plasmodium falciparum* is the species that causes the most life-threatening form of malaria. The disease is transmitted to a person by a bite of an infected female *Anopheles* mosquito [18]. It can also be transmitted from one person to another through blood transfusion, an organ transplant, and sharing of needles or syringes [18,19]. It can also be transmitted from an infected mother to a child at birth. This disease is common in the tropical and subtropical regions across the world, which include sub-Saharan Africa, Asia and Latin America [19,20]. Malaria has a major negative impact on economic development, thus leading to poverty [21]. The symptoms usually begin about ten to fifteen days after a mosquito bite [21]. Typical, symptoms of malaria include: fever, headaches, and vomiting, but in severe cases it can cause seizures, anemia, abnormal pains and coma [22].

### 2.2. Leishmaniasis

Leishmaniasis is caused by the *Leishmania* parasite that usually lives in infected sand flies. It can be transmitted from a bite of a female infected sand fly [23]. This disease can also be transmitted from one person to another through blood transfusion or by sharing of needles [24]. Leishmaniasis is found in parts of tropic and subtropical regions which include: East Africa, South America and Asia [25]. There are several different forms of leishmaniasis but the most common in humans are: cutaneous and visceral [26]. The main symptom of cutaneous leishmaniasis is skin sores. Common symptoms for visceral leishmaniasis are weight loss, fever, enlarged spleen and enlarged liver [27].

### 2.3. Schistosomiasis

Schistosomiasis is the third most devastating parasitic disease in the world [28]. It is the cause of mortality and morbidity in developing countries such as Africa, South America and Asia [29]. In 2014, an estimated 61.6 million people were infected with schistosomiasis [30]. It is caused by a parasite called *Schistosoma* and the parasite is a fluke [31]. The parasite affects the intestines and bladder, but because it lives in the blood, thus it can also affect other systems. After infection, the person may develop a rash or itchy skin within 1–2 months with symptoms such as muscle aches, fatigue, cough, weight loss, fever and chills [32].

### 2.4. Toxoplasmosis

Toxoplasmosis is an infection caused by a parasite called *Toxoplasma gondii* [33]. The infection usually occurs from the exposure to infected cat faeces, by eating undercooked meat, or it can be transmitted from mother to child during pregnancy [33]. It can also be found in contaminated water [34]. People who are at a high risk of the infection are those with compromised immune systems and infants born to mothers with active infection during pregnancy [35]. Toxoplasmosis can cause serious complications to those with weakened immune systems such as infants, pregnant women and people living with HIV/AIDS [35]. Most healthy people who are infected with toxoplasmosis show no signs or symptoms but some may develop symptoms similar to flu, fever, body aches, headache and fatigue [36]. Toxoplasmosis is life-threatening in people with low immune systems and they are at risk of developing seizures, confusion, poor eye vision, and lung infection [36,37].

## 3. Viral Infection

Viruses are microorganisms that replicate only within living cells by using the cells’ enzyme systems [38]. They cause diseases such as HIV, herpes, cervical cancer (HPV), meningitis, hepatitis, and influenza etc. [39].

### 3.1. HIV

HIV is a human immune virus that causes acquired immunodeficiency syndrome (AIDS) over time [40]. HIV attacks immune system of the body, causing low CD4 count. AIDS is the final stage of HIV infection, but not everyone with HIV develop AIDS [41]. When HIV has manifested into AIDS, it becomes life-threatening by destroying the white blood cells that usually fight infections [42]. This can cause serious infections and diseases like tuberculosis, candidiasis and meningitis etc. [43]. HIV is transmitted through sexual intercourse with an infected person, through blood transfusion from an infected person, and from an infected mother to her baby through breastfeeding, during pregnancy [44]. The symptoms of HIV usually develop several months or even years after infection with the virus [45]. The early symptoms of HIV infection include: fever, chill, joint pain, and rashes. If HIV has manifested into AIDS, then the symptoms may include diarrhea, dry cough, weight loss, night sweats and serious fever [45,46].

### 3.2. Influenza

Influenza is a contagious respiratory infection which affects people of all ages [47]. The virus is transmitted through the air by coughs and sneezes and infects the nose, throat, mouth, and lungs. It can also be transferred by touching surfaces that are already contaminated with the virus [48]. The virus can be deadly in individuals with low immune system (i.e., newborn babies, elderly people and people living with chronic diseases) [49]. There are three common forms of influenza, namely: type A, B and C [50]. Type A flu viruses are the most dangerous and exhibit deadly complications and are responsible for the large human influenza pandemics [51]. The virus mostly affects humans but animals and wild birds are also known hosts for the flu virus [51]. Type B virus is less common than A and it only affects humans [52]. Although type B is very harmful, but it is less severe than type A [53]. Type B influenza does not cause human pandemics [54]. Influenza type C causes mild diseases and is less common than other types, there are no epidemics associated with influenza type C [55]. Symptoms include cough, chills, headaches, sore throat and body muscle aches [56].

### 3.3. Meningitis

Meningitis is a viral infection of the meninges, the tough layer of tissue surrounding the spinal cord and the brain [57]. Meningitis can lead to brain swelling causing permanent disabilities such as coma, and can lead to death if not treated [58]. There are four common type of meningitis which include bacterial, viral, fungal and aseptic meningitis [59]. Bacterial meningitis is the most life-threatening and can lead to death in few hours [59]. It is caused by the bacterial such as *Streptococcus*, *Streptococcus pneumonia*, *Neisseria meningitides* and *Listeria monocytogenes* [60]. Most people are lucky to recover from it, but they are likely to get permanent disabilities such as hearing loss, brain damage and coma [60]. Viral meningitis is caused by viruses such as enteroviruses, herpes varicella and mumps viruses [61]. Fungal meningitis is caused by pathogens such as *Candida spp.-Histoplasma capsulatum* and *Cryptococcus neoformans*. Fungal meningitis is most common in people with low immune systems, and it is more severe in people with impaired immune systems (e.g., organ transplantation) [62]. Parasitic meningitis is caused by parasites such as *Angiostrongylus cantonensis*, *Schistosoma*, *Toxocariasis* and *Gnathostoma spinigerum*. The infection is believed to occur when there is a predominance of eosinophilia in the CSF [63]. Symptoms of meningitis include fever, stiffness of neck, nausea, headache and vomiting [64].

### 3.4. Herpes

Herpes is a sexually transmitted disease [65]. Oral herpes is also known as HSV-1, or type 1 herpes simplex [66]. It can be transmitted via infected saliva, mucous membranes or skin [67]. This virus causes sores in the mouth, gums, tongue, face or nose. It causes symptoms such as fever, swollen lymph nodes and muscle aches [68]. Genital herpes is also known as HSV-2, or type 2 herpes simplex [69]. This virus causes sores around the genital areas. The virus is transmitted through skin-to-skin contact with sores [69]. Genital herpes is most likely to affect women than men and women with HIV are difficult to treat resulting in the administration of higher doses of antiviral drugs [70]. The virus sometimes hides in the nerves causing no symptoms. If the symptoms are visible, then a person may experience itchy painful blisters which could results in ulcers [71].

### 3.5. Hepatitis

Hepatitis is the inflammation of the liver tissue resulting from alcohol abuse, certain medications and toxins [72]. Common types of hepatitis are Hepatitis A, B and C. Hepatitis A is a virus that causes liver infection [72]. It is transmitted through the digestion of food or water that is already contaminated with the faeces of an infected person [73]. Symptoms of hepatitis A include vomiting, tiredness, joint pains, dark urine and intense itching [74]. Hepatitis B is caused by the virus hepatitis B and it is transmitted from blood and body fluids of an infected person. It is also transmitted from mother-to-unborn baby if the mother is infected [75]. Hepatitis B can be prevented by vaccination. Symptoms are similar to those of type A; they include headache, dark urine, and vomiting etc. [76]. Hepatitis C causes serious liver cancer, which could lead to liver transplant [77]. Approximately 80% of patients with hepatitis C develop chronic liver infection [78]. It is transmitted via sharing needles with an infected person, through ingesting drugs and through mother to child transmission during pregnancy. Symptoms include fever, fatigue, nausea, abdominal pain and jaundice [79].

### 3.6. Cervical Cancer

Cervical cancer is caused by a virus called human papillomavirus (HPV) and this virus causes the growth of abnormal cells on the cervix which is cancerous [80]. HPV is transmitted via sexual intercourse [81]. There are factors that contribute to the development of HPV, such as having many sexual partners, people living with HIV I are likely to be infected with the disease, long-term use of contraceptives, having several pregnancies and giving birth at a young age [82]. Symptoms of cervical cancer include abnormal vaginal bleeding, abnormal vaginal discharge, and vaginal bleeding after menopause, heavy periods and vaginal bleeding after sex [83].

## 4. Application of Dendrimers in the Treatment of Infectious Diseases

Infectious diseases are caused by microorganisms, such as bacteria, viruses, parasites or fungi [84]. Infectious diseases are currently being treated by therapeutics such as antibiotics, antiviral, anti-parasitic and antifungal [85]. Most of these therapeutics suffer from severe limitations such as drug resistance, toxicity and their routes of administration result in poor patient compliance [86,87]. The drug-resistant problem is due to different mechanism such as increased efflux system; reduced membrane permeability or increase of drug degradation [88]. Due to these limitations, the application of targeted drug delivery system is an attractive carrier for the treatment of infectious diseases [89]. Drug delivery system is used to transport pharmaceutical compounds directly to the targeted organs or tissue with less toxic effects on the organs/tissue [90]. There are many different types of drug delivery systems, such as dendrimers, micelles, liposomes, nanospheres, nanocapsules, hydrogels and polymer-drug conjugates [91]. An ideal drug delivery system must be able to reduce drug toxicity, improve bioavailability, biocompatibility, enhance drug solubility, non-immunogenic, biodegradable, enhance patients’ compliance and be able to overcome drug resistance [92,93].

**Figure 1:**
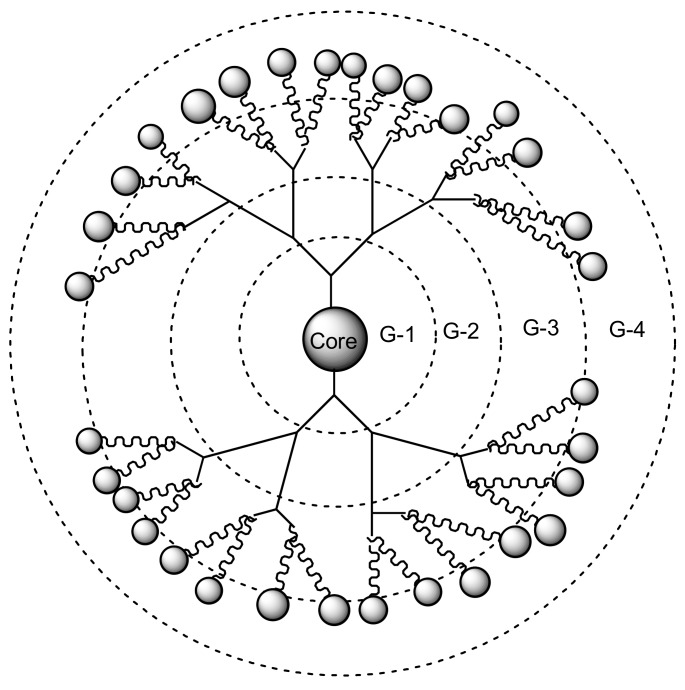
Schematic diagram of dendrimers (G1–G4).

Dendrimers are synthetic polymers with three-dimensional, star-shaped and branched macromolecules [94]. They are made up of the interior layers and the exterior layers. The exterior layer is composed of functional groups which are useful for conjugation of drugs and targeting moieties [95]. The interior layers are suitable for encapsulation of drug molecules with improved drug efficacy, reducing drug toxicity and control release mechanisms of drugs [96]. They are water-soluble, biocompatible, polyvalence, and biodegradable [96]. These properties make them useful for drug delivery and they are being investigated by several scientists [97]. Figure 1 shows a schematic diagram of dendrimers from generation one to four. This review will demonstrate the importance of dendrimers as a targeted delivery system for the treatment of infectious diseases precisely viral and parasitic infections.

### 4.1. Various Dendrimers and Their Applications

**Figure 2:**
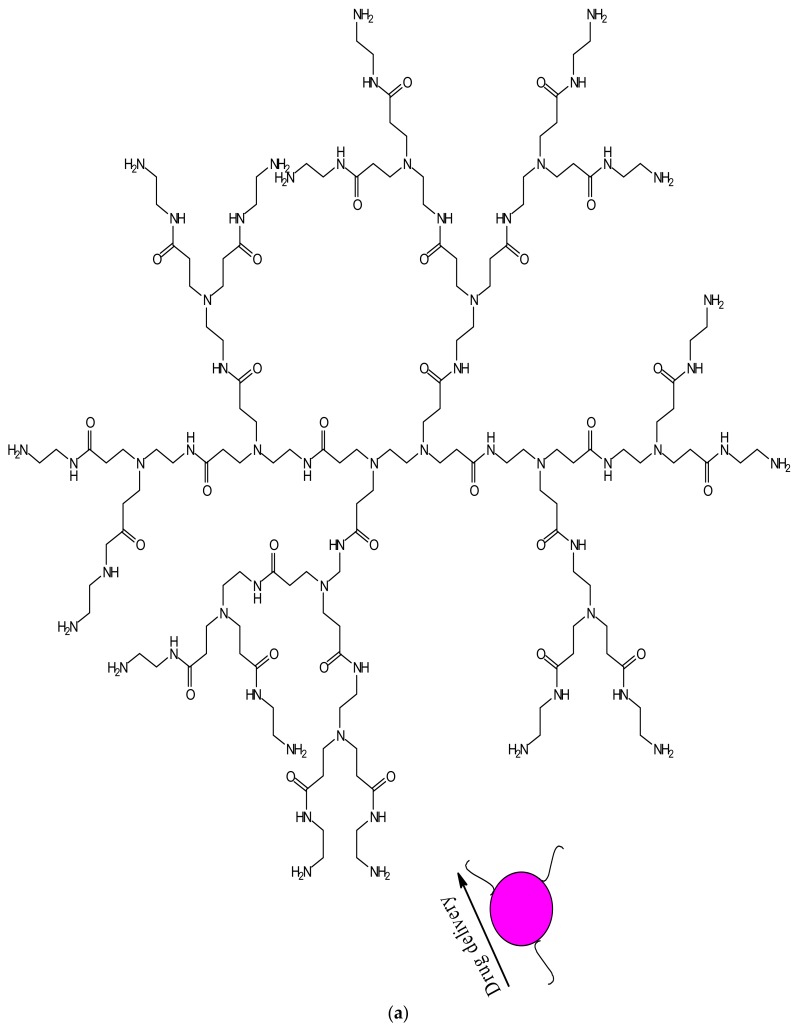

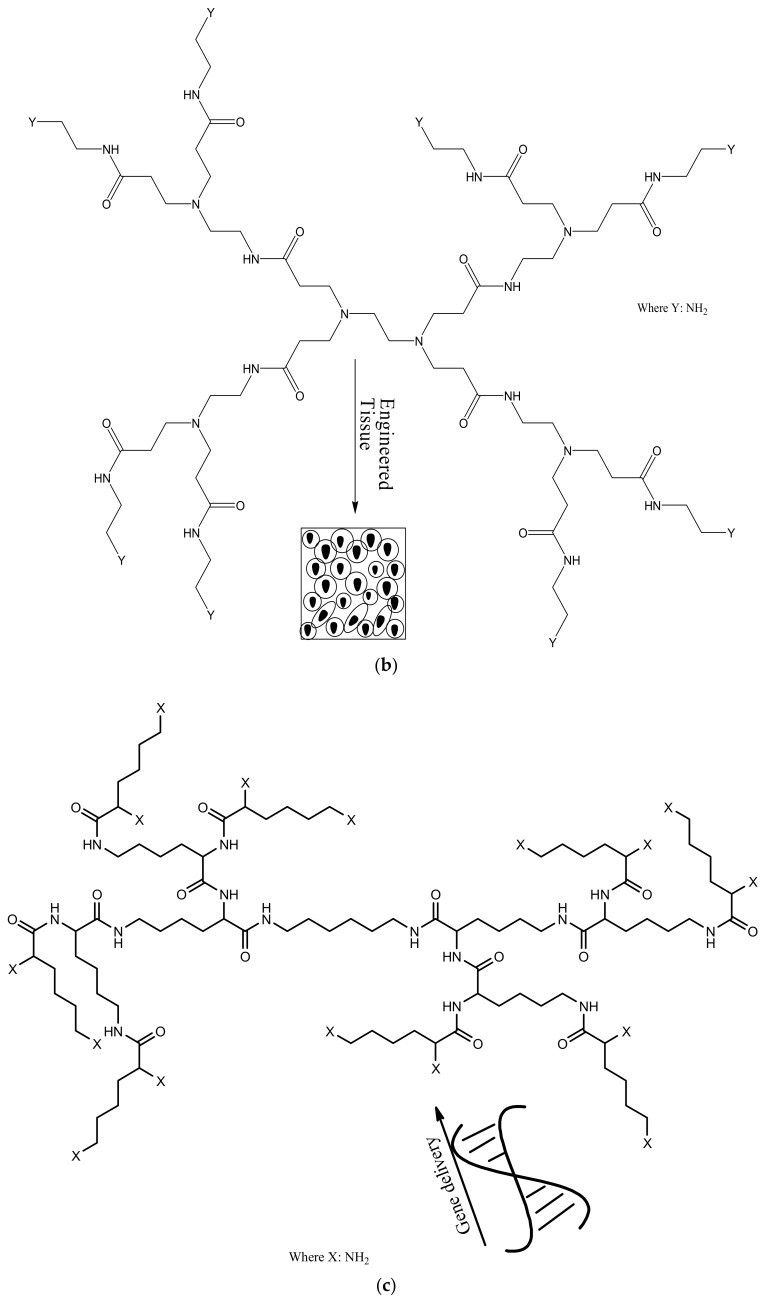

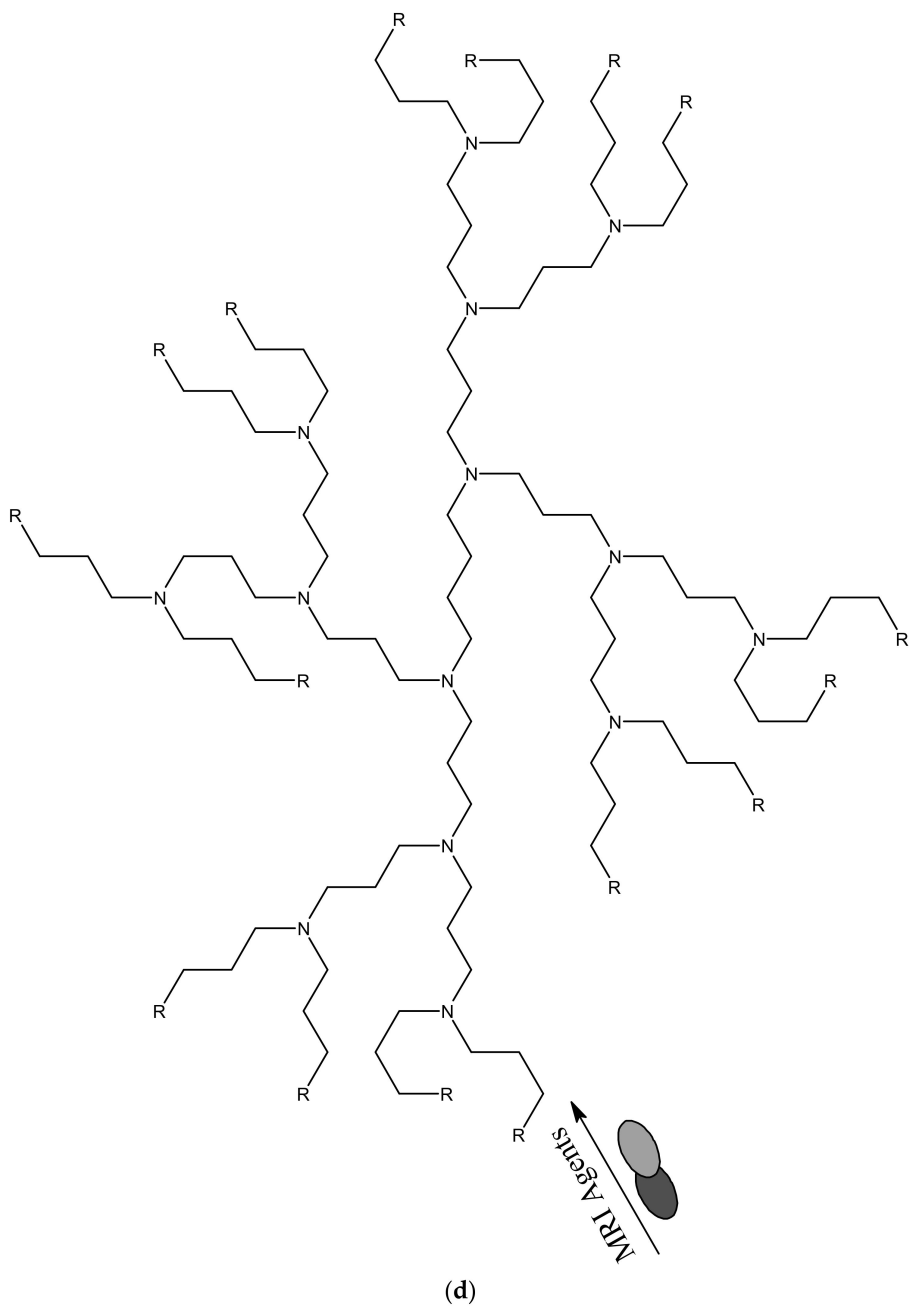
Schematic representation of dendrimers and their applications, (**a**) PAMAM-G3 dendrimers for drug delivery; (**b**) PAMAM-G1 dendrimers for tissue engineering; (**c**) PLL dendrimers for gene delivery; and (**d**) PPI dendrimers for diagnosis.

Dendrimers have several medical and practical applications; they can be used for drug delivery, gene delivery, tissue engineering and for diagnosis [98]. Several dendrimers have been developed for biomedical applications [98]. Polyamidoamine (PAMAM) has been used extensively for drug delivery (Figure 2a) and tissue engineering (Figure 2b), because of their biocompatibility, hydrophilic nature and non-immunogenic effect [99]. PAMAM dendrimers consist of ethylenediamine core, their branching units consist of amine groups that can be used to load drugs, antibodies, enzymes and other bioactive agents [100,101]. Poly-l-lysine (PLL, Figure 2c) dendrimers are mostly used as gene carriers; they contain two primary amines which are often modified to enhance their therapeutic effects [102]. Poly-l-Lysine dendrimers are biocompatible, flexible, biodegradable and water-soluble [102]. Poly (propylene imine) (PPI, Figure 2d) dendrimers are used for diagnosis [103]. The core of PPI is usually based on a 1,4-diaminobutane or ethylenediamine and the branching units consists of propylene imine monomers [103].

### 4.2. Dendrimers for the Treatment of Leishmaniasis

**Figure 3:**
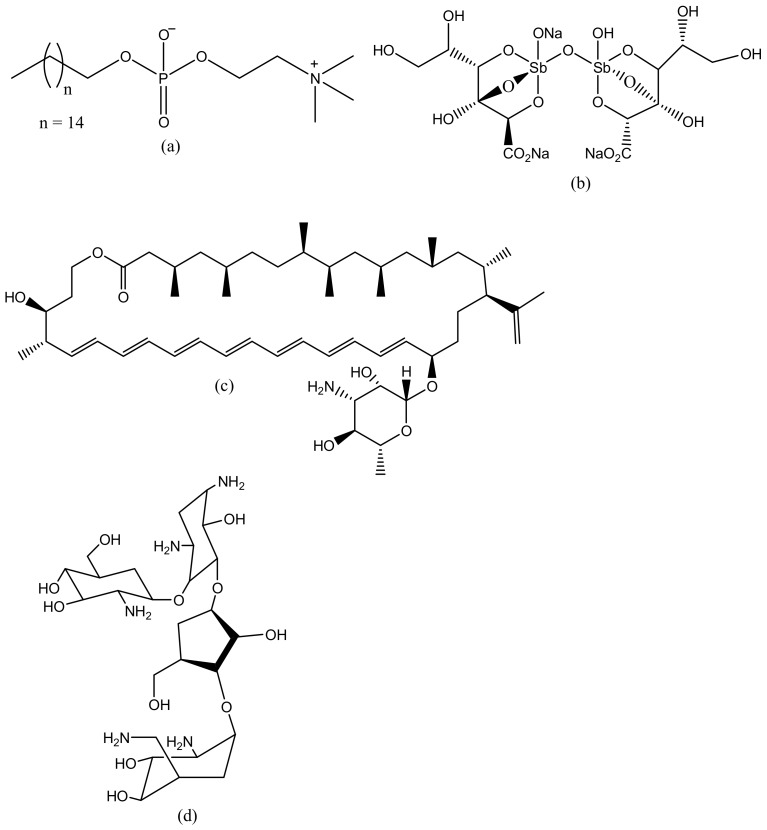
Antileishmaniasis drugs (**a**) Miltefosine; (**b**) Sodium stibogluconate; (**c**) Amphotericin B; (**d**) Paromomycin.

**Figure 4:**
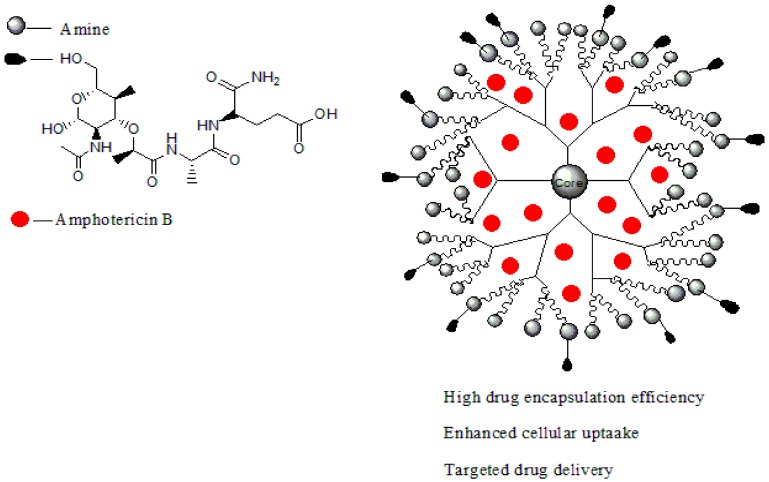
Dendrimer loaded with amphotericin B.

Leishmaniasis is a life-threatening disease that is caused by a *Leishmania* parasite and transmitted through a bite of an infected sand-fly [104]. About 12 million cases of people are affected by leishmaniasis across the world and two million cases of leishmaniasis occur annually [105]. For several decades, leishmaniasis was being treated with drugs such as sodium stibogluconate (Pentostam, Figure 3b) and meglumine antimoniate (Glucantime) [106]. Although, these drugs have been used for several decades, they have been reported to develop resistance to leishmaniasis with side effects such as cardiotoxicity and pancreatitis [107]. A study in India revealed that about 65% of patients relapsed after treatment with an antimonial [108]. There is an increase in the cases of resistance to pentavalent antimonial reported worldwide [108,109]. Due to emergence of resistance of the parasite to antimonial drugs, amphotericin B (AmB, Figure 3c), Miltefosine (Figure 3a), and paromomycin (Figure 3d) are being used as alternative therapeutics for the treatment of leishmaniasis [110]. However, there are severe side effects associated with their use and they are also expensive [111]. Amphotericin B is used as both antifungal and antiparasite, although it shows a good efficacy, but it is expensive and requires oral dose [112]. Miltefosine is an anticancer drug but it has been approved as an oral drug for leishmaniasis [113]. Miltefosine also shows good efficacy, but it is very expensive with limitations such as low blood platelets, nephrotoxicity, diarrhea etc. [113]. Recently, the use of nanocarriers such as dendrimers have shown promising results in the treatment of leishmaniasis [114]. Dendrimers have the ability to transport drugs to the targeted site, reduce drug toxicity, increase drug solubilisation, protect the drug from degradation and ultimately kill the protozoa [115]. Jain et al. developed a formulation of muramyl dipeptide conjugated with poly (propyleneimine) (PPI) dendrimers encapsulated with amphotericin B (Figure 4). The synergistic antiparasitic activity of the formulation was evaluated in vivo. The in vivo results showed that the formulation was active against the parasite infection of macrophage cell lines and balb/c mice. The toxicity of the formulated drug loaded dendrimers was compared to the marketed formulation of amphotericin B. The prepared formulation exhibited a reduction of (*p* < 0.01) in toxicity towards human erythrocytes cells and J774A.1 macrophage cells [116], revealing the potential of the dendrimers to reduce the toxicity associated with amphotericin B (Table 1). The macrophage targeting ability of the formulation was enhanced, resulting in the killing of the parasites. These results suggested that the formulations are potential immunomodulatory with antileishmanial activity for targeted drug delivery of amphotericin B. Daftarian et al. developed a complex between liposome amphotericin B and Pan-DR-binding epitope-based dendrimers to study the therapeutic efficacy of low dose LAmB/PDD against full dose of LAmB via *L. major* mouse method [117]. The formulation exhibited reduced toxicity which was visible by dose reduction. In vitro toxicity of the formulation revealed reduced toxicity on Hep2 cells. The formulation was also delivered selectively to parasite reservoir cells, phagocytes [117]. The in vitro and in vivo studies revealed an 83% improvement in drug efficacy with a significant reduction of parasite burden and toxicity. Jain et al. prepared a poly (propylene imine) dendrimers containing mannose loaded with amphotericin B (Table 1). The formulation exhibited good drug incorporation efficiency and the in vitro results revealed pH-dependent drug release mechanism. The formulation also exhibited reduced toxicity on human erythrocytes and macrophage cells and the efficacy of the loaded drug was not compromised [118]. These dendrimers were observed to have significant antiparasitic activity towards *L. denovani* amastigotes with a promising antileishmanial activity [118]. Furthermore, pharmacokinetic and organ distribution studies revealed the controlled delivery mechanism of the formulation which was characterized by an enhanced drug uptake in macrophage-rich organs.

### 4.3. Toxoplasmosis

**Figure 5:**
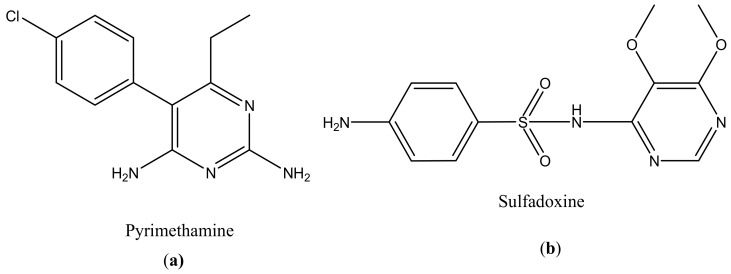
Anti-toxoplasmosis drugs: Pyrimethamine (**a**); Sulfadoxine (**b**).

About two billion people are infected by *Toxoplasma gondii*. This parasite causes morbidity and mortality [123]. Pyrimethamine (Figure 5a) and sulfadoxine (Figure 5b) are currently being used for the treatment of toxoplasmosis (Table 1). However, there are some limitations in their use, such as toxicity and hypersensitivity [119]. The main problem with the drugs is that they do not eliminate the parasite because *Toxoplasma gondii* encysted bradyzoites [124]. There is a pressing need to develop a new strategy that can effectively treat *toxoplasma gondii* infection by crossing the host cell membrane, the parasitophorous vacuole, and the tachyzoite membranes [124]. Transductive peptide dendrimers are potential therapeutics because they can transport small bioactive molecules across multiple membranes through intracellular tachyzoites and encysted bradyzoites and they can also enhance the toxicity of the drugs [125]. There are few studies that have revealed the importance of dendrimers in the treatment of *Toxoplasma gondii* [126].

**Figure 6:**
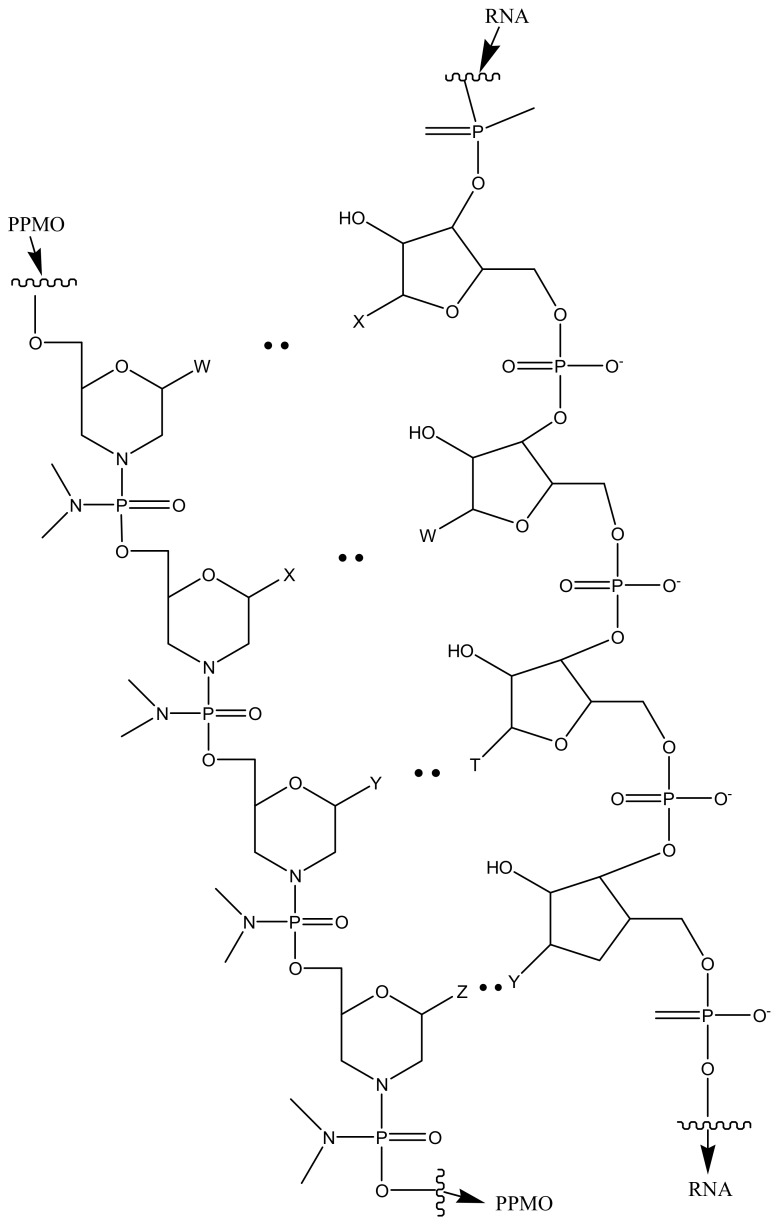
Schematic representation of PPMO with transductive peptide.

Lai et al. evaluated the potential of the treatment of *Toxoplasma gondii* infection by conjugating phosphorodiamidate morpholino oligomers (PPMO) with transductive peptide [127]. The formulation reduced transfected *T. gondii*’s fluorescence, luminescence and limited tachyzoite replication. In vivo studies on infected mice revealed the reduction in the number of viable parasites after administration [127]. Figure 6 is a schematic presentation of PPMO with transductive peptide dendrimers.

Prieto et al. prepared poly (aminoamine)-based anionic and cationic dendrimers containing a reduced dose of sulfadoxine (0.03–33 mM). The MTT results on Vero and J774 cells showed no toxicity for cationic-sulfadoxine complex incubated between 0.03 and 33 mM of dendrimers concentration. However, the anionic-sulfadoxine complex resulted in enhanced cytotoxic effects when incubated at higher than 33 mM of dendrimers concentration. Both dendrimers were further tested in vitro using Vero infected cells with RH strain of *Toxoplasma gondii* for a period of 4 h in treatment. Cationic dendrimers produced the highest infection decrease of 60% at 0.03 mM and anionic dendrimers produced between 25% and 40% reduced infections. These results suggest that a nano dose of sulfadoxine- cationic complex can be used as a potential anti-toxoplasmic therapy [128]. The dendrimer exhibited high antiparasitic effect even when administered at very low doses over a period of 4 h of treatment. This revealed that the dendrimers have an antiparasitic effect. The dendrimers antiparasitic effects are attributed to a combination of surfacial activity and endosomolytic effect.

### 4.4. Schistosomiasis

**Figure 7:**
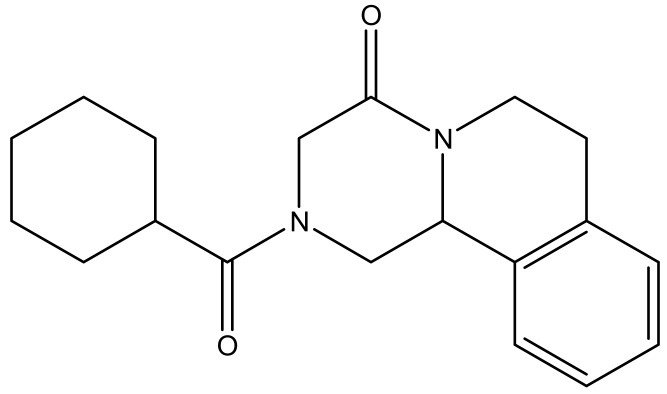
The anti-schistosomiasis drug: Praziquantel.

Schistosomiasis is still a major problem in the world; about 200 million people are infected with schistosomiasis across the globe. The most infected countries are Africa, Asia and South America [129]. The disease is caused by numerous species of trematodes from the genus *Schistosoma* [130]. *Schistosoma* is treated with praziquantel and is an effective bioactive agent (Figure 7) [131]. Despite its availability and cost-effectiveness, it does not prevent relapse [131]. The emergence of resistance of praziquantel to schistosomes is spreading and causing a major concern and there is a need to develop a new vaccine to treat *Schistosoma* [132]. Dendrimers have been investigated by several researchers and they are promising therapeutics to eliminate the disease. Sikwal et al. investigated amphiphilic dendrimers potential applications for pharmaceutical and biomedical applications [133].

Wang et al. designed PAMAM dendrimers for the delivery of schistosomiasis japonica DNA vaccine and investigated its ability to enhance a protective effect against *Schistosoma japonicum* infection. The dendrimers were prepared by a Lysine-Modified method to form PAMAM-Lys. The dendrimers cytotoxic effects on 293T cell lines were evaluated by MTT assay, while Poly-lysine (PLL) was used as a control. It was observed that increasing PLL concentration decreased cell viability. Overall, the dendrimers exhibited 80–90% cell viability, showing no genitive effect on it cytotoxicity. When PAMAM-lys was combined with DNA vaccine (Table 1), it exhibited a higher level of efficacy when compared to the free DNA with reduced worm infection by 45–50% and 59–62% liver eggs reduction. These results showed that DNA vaccine with the novel PAMAM-lys dendrimers can enhance immunoreactivity of DNA vaccine, and can be used for the prevention of *S. japonicum* infection [120]. The formulation enhanced IgG2a antibody response with an increase in the production of IL-2 and IFN-γ.

### 4.5. Malaria

**Figure 8:**
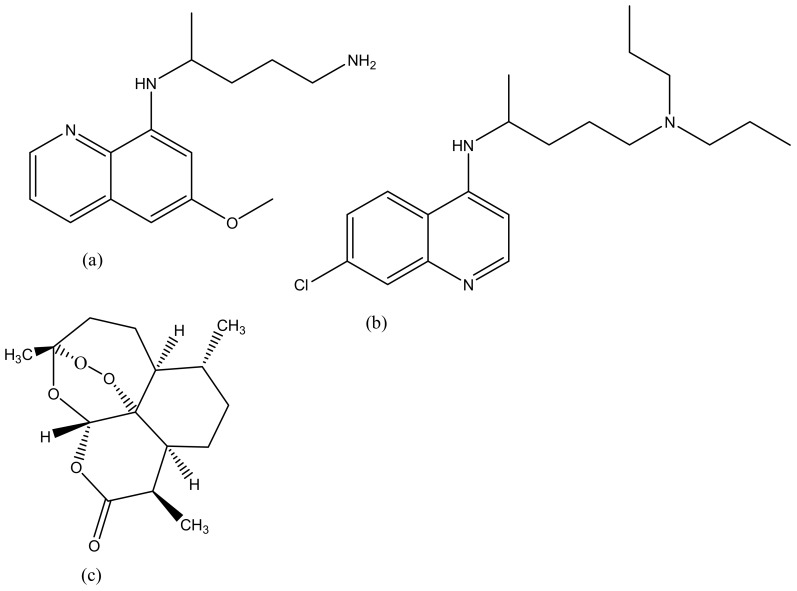
Antimalarials drugs: Primaquine (**a**); Chloroquine (**b**); Artemisinin (**c**).

**Figure 9:**
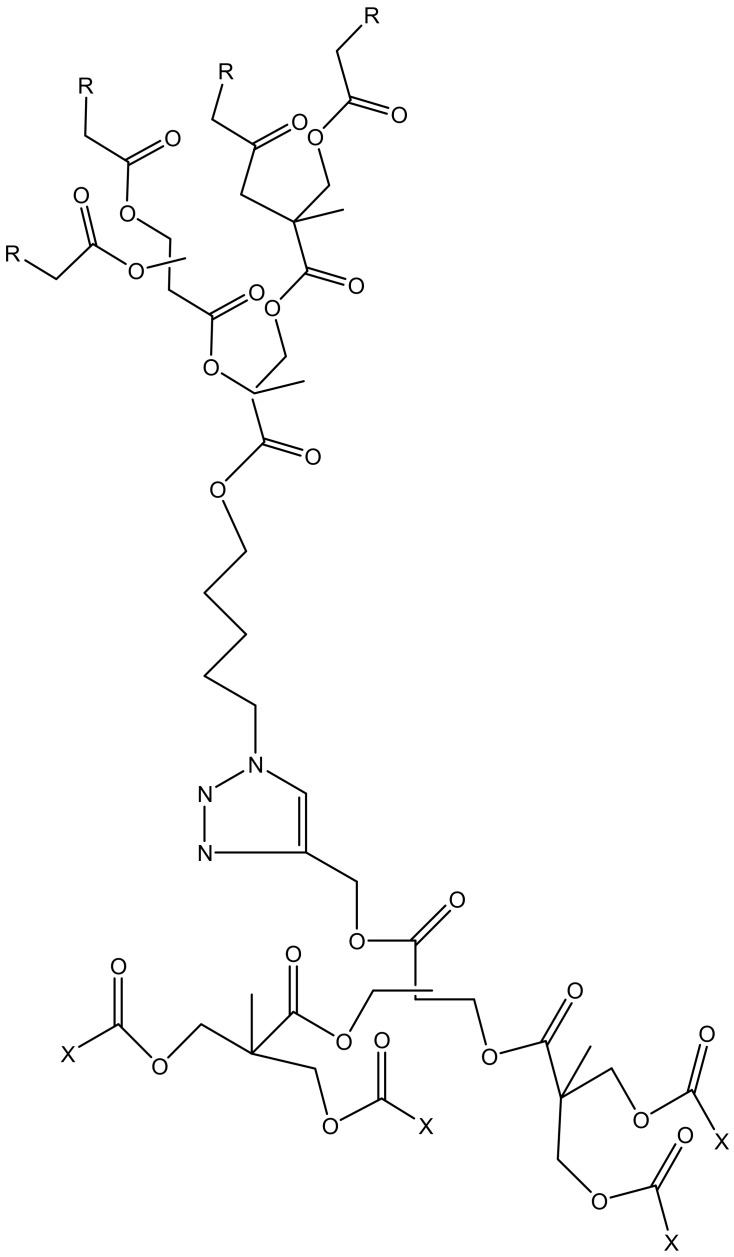
Schematic presentation of Amphiphilic dendrimers (R = C_17_H_35_, X = H_2_N).

Malaria is life-threatening and half of the world’s population is at risk of malaria transmission [134]. People that are at a higher risk of being infected by malaria are children under the age of 5 years, pregnant women, people living with HIV/AIDS and low-immunitive travellers from malaria-endemic regions [135]. In 2015, 214 million cases were reported worldwide, with most deaths reported in sub-Saharan Africa, South-East Asia and the Eastern Mediterranean [136,137]. Malaria is treated using antimalarials such as chloroquine (Figure 8a), primaquine (Figure 8b), artemisinin (Figure 8c) and its derivatives. However, they suffer from severe drug resistance and toxicity which results in treatment failure [137]. Due to the emergence spread of drug resistance, drug toxicity and poor patient compliance, there is a need to develop drug delivery systems that can overcome drug resistance, reduce toxicity and improve patient compliance [138]. Dendrimers are promising delivery systems that have been used by many researchers due to its excellent biocompatibility and biodegradability [139]. Movellan et al. synthesized dendritic derivatives based on 2, 2-bis (hydroxymethyl) propionic acid (bis-MPA) and Pluronic polymers containing chloroquine and primaquine (Table 1). They were investigated for their targeting ability in *Plasmodium*—infected red blood cells (pRBCs) and their antimalarial activity against the human pathogen *Plasmodium falciparum* and in vivo against the rodent malaria species *Plasmodium yoelii*. From the in vitro results, the dendrimers exhibited antimalarial activity with reduced IC_50_ of chloroquine and primaquine by 3- and 4-fold down to 4.0 nm and 1.1 μm, respectively. The dendrimers were also found to exhibit specific targeting mechanism to the pRBCs when compared to non-infected RBCs. Amphiphilic bis-MPA derivatives- based dendrimers have been used in the application of biomedical field (Figure 9). Bis-MPA derivatives have shown a great therapeutic efficacy in drug delivery because of their ability to be degraded by enzymes, their compatibility, and high solubility in biological environments. They also consist of functional groups that make it easy to encapsulate antimalarial drugs. Figure 7 shows a typical example of amphiphilic dendrimers [121].

Agrawal et al. synthesized coated and uncoated poly-l-lysine dendrimers having polyethylene glycol (PEG-100) as a core for the delivery of chloroquine phosphate (Table 1). The in vivo results revealed that the dendrimers exhibited controlled drug release mechanism. The coated drug dendrimers exhibited reduced haemolytic toxicity when compared to the free drug [122]. The uncoated and coated dendrimers were synthesized by the protection and deprotection steps of l-lysine by di-BOC (di-tertiary butyl pyrocarbonate). The ex vivo results of both the uncoated and coated dendrimers revealed that the formulations were 5 times effective in reduction of phagocytosis for the coated dendrimers. The dendrimers were also found to exhibit controlled drug release mechanism. These findings suggested that the coated dendrimers were less immunogenic than the uncoated formulations.

## 5. Application of Dendrimers for the Treatment of Viral Infections

### 5.1. HIV

**Figure 10:**
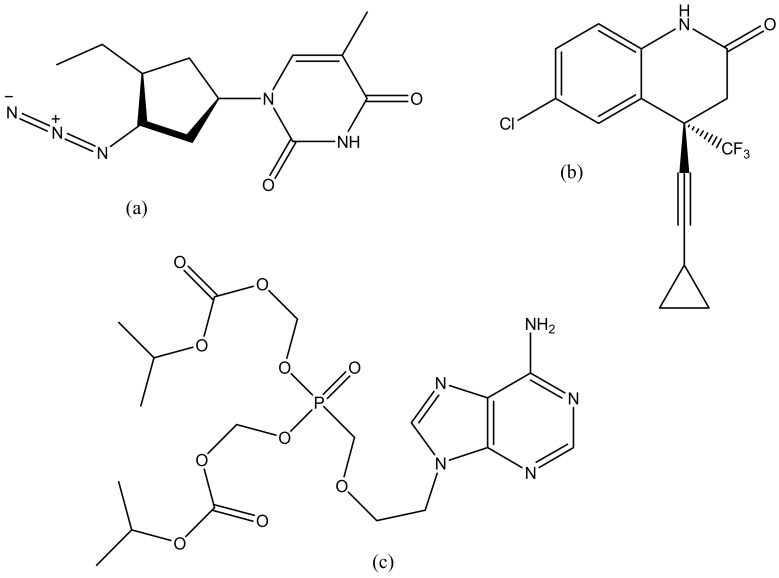
Antiretroviral drugs: Efavirenz (**a**), Zidovudine (**b**), Tenofovir (**c**).

According to the latest data in 2012, the number of human immunodeficiency virus (HIV) infections has decreased by 35%. However, 2.3 million people are infected with HIV with high death rates occurring worldwide [140,141,142]. 60% of people with HIV contracted the virus during sexual intercourse [143]. Therefore, AIDs are still a serious problem across the globe and there is a need to develop a new strategy to eliminate this virus [143]. The use of antiretroviral drugs (ARVs) is effective against HIV infection by delaying the disease progression as well as mortality rate in HIV-infected patients [144]. Although these antiretroviral are effective, they do not cure or eliminate the virus; therefore, there is a need for a new strategy [145]. Currently, nanotechnology provides novel nanoparticles such as dendrimers that can transport antiretroviral to the desired organs. Dendrimers have an exterior layer that is dominated by functional groups useful for the conjugation of drugs and targeting moieties [146]. The interior layers are suitable for the encapsulation of drug molecules with improved drug efficacy, reduced drug toxicity and controlled release mechanisms. Combination therapy is one promising method to fight this disease [147]. Cardoba et al. developed a polyanionic carbosilane dendrimers 9G3-S16 and G2-NF16) containing zidovudine (Figure 10a), efavirenz (Figure 10b) and tenofovir (Figure 10c) as anti-HIV-1 microbicides (Table 2). These dendrimers were tested against X4 and R5 HIV-1 strains in vitro. The prepared dendrimers showed a synergistic activity profile against both strains, and in human cells. The sulphated and naphthylsulfonated carbosilane dendrimers were able to inhibit viral infection by blocking the interaction between gp120 and CD4. This means that carbosilane dendrimers can block HIV infection at different stages of the HIV-1 life cycle before viral integration. The dendrimers act by electrostatic interactions with the viral envelope proteins resulting in the blockage of gp120/CD4 interaction and avoiding viral entry [148].

Zidovudine has been reported to be a very effective antiretroviral drug in the treatment of HIV virus. However, it has been reported to suffer from pharmacological limitations such as poor bioavailability, short half-life, and resistance. In order to overcome these limitations, Jain et al. developed a sustained release formulation of poly (propyl ether imine) dendrimers for the delivery of zidovudine (Table 2). Results from FTIR and NMR shows that zidovudine was successfully encapsulated onto dendrimers. Cumulative drug release of zidovudine from the dendrimers was 6.5 ± 0.3% when compared to the 95.8 ± 4.1% release from the control drug solution, hence revealing the sustained release profile of the dendrimers. The dendrimers also showed a reduction in the haemolytic toxicity due to the stable drug encapsulation in the dendrimers when compared to pure zidovudine drug solution. These findings suggest that the dendrimers are potential carriers for sustained delivery of zidovudine [160].

**Figure 11:**
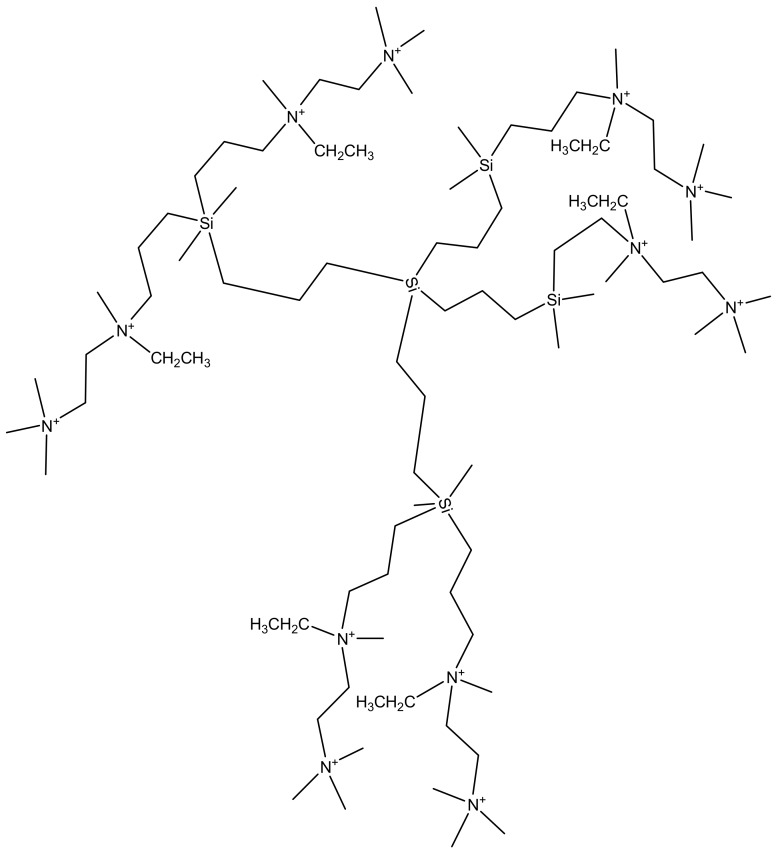
Second generation cationic carbosilane dendrimers branched with carbon-silicon bonds.

Crespo et al. formulated carbosilane dendrimers conjugated with tenofovir and maraviroc for the treatment of HIV-1 infection (Table 2). They were evaluated for anti-HIV-1 activity, cytotoxicity and vaginal irritation effects. The combination of maraviroc and tenofovir into the dendrimers exhibited a greater anti-HIV-1 activity than a single drug. These dendrimers were found to exhibit a greater synergistic activity profile due to the weighted average combination indices varied between 0.06 and 0.38 [153]. No vaginal irritation was detected in the female BALB/c mice. These results suggest that combination of two or three drugs into dendrimers can increase the antiviral activity. Telwatte et al. developed dendrimers SPL7013 as topical microbicides for the prevention of the transmission of human immunodeficiency virus [164]. It was prepared in a mucoadhesive carbopol gel. The formulation exhibited HIV-1 virucidal activity against X4 and R5X4. The mode of action of the formulation on X4 strain virus was via irreversible binding to HIV-1 envelope proteins. The inhibition of R5 strains was via reversible binding to HIV-1 envelope proteins, host cell CD4 and chemokine receptors [164]. Chonco et al. also prepared dendrimer-based microbicides which were water-soluble against HIV infection (Table 2). The formulation blocked activated primary peripheral blood mononuclear cells (PBMC) infection with HIV-1 and HIV-2 strains, inhibited partially HIV crossing through trans-epithelial monolayer in vitro. The mechanism of inhibition of the formulation against HIV-1 and HIV-2 is attributed to direct viral inactivation by blocking the CD4 receptor at the surface of the target cells. The interaction of the anionic charges of the formulation to HIV gp120 was higher in HIV-1 strains than in the HIV-2 strains due to variation in amino acids in the gp120 region [158]. Han et al. prepared polylysine-dendritic sulfated cellobiose via condensation of polylysine dendrimer generation 3 with acetylated cellobiose followed by deacetylation and sulfation. The sulfated cellobiose dendrimer exhibited anti-high HIV activity as dideoxycytidine, an anti-HIV drug and this is attributed to their cluster effects which improves their interaction with proteins on the surface of the viruses [165]. Borges et al. covalently attached globotriose and 3′-sialyllactose carbohydrate head groups found on two glycosphingolipids to a dendrimer core. The formulation inhibited HIV-1 infection of T cell lines and primary peripheral blood mononuclear cells (PBMC) by T cell line-adapted viruses or primary isolates, with IC_50_ 0.1–7.4 μg/mL [166]. Doménech et al. revealed that gallic acid-triethylene glycol dendrimers can bind to the C-terminal domain of capsid protein. The dendrimers with large hydrophobic moiety at the dendritic branching inhibited the assembly of the human immunodeficiency virus capsid in vitro revealing the potential of dendrimers as anti-HIV drugs for targeting capsid assembly [167]. Price et al. studied the retention of HIV-1 and HSV-2 inhibitory levels of SPL7013 gel in female genital tract over a period of 24 h. 9 and 2.5 mg of SPL7013 administered resulted in high level of inhibition of HIV-1 and HSV-2, respectively [168]. HIV-1 and HSV-2 inhibition was maintained in 6/11 women. The formulation did not induce vaginal, vulvar or cervical irritation [168]. Carbosilane are great candidate for the delivery of HIV-peptides. They form stable compounds with nucleic acids and protect them from binding to proteins. These dendrimers were reported in the study by Lonov et al. The formulation was prepared in molar ratio (2.5–3):1 of dendrimer: peptide with size range of 180–275 nm and positive surface charge. The dendrimers were terminated with amino groups representing cationic particles that are suitable for binding the negatively charged HIV derived peptides and for the delivery of HIV peptides to dendritic cells. Figure 11 shows a second generation of carbosilane dendrimers [169]. De Las et al. prepared water-stable carbosilane dendrimers as non-viral vectors for transfecting nucleic acids against HIV. These systems formed nanoconjugates with nucleic acids revealing good interaction between the dendrimers and the nucleic acid. The degree of transfection using these nanoconjugates ranged between 70–90% depending on the generation [170]. Jiménez et al. developed dendrimers as a delivery vector for anti-HIV drugs that is capable of crossing the blood-brain barrier (BBB). A time-controlled degradation of the dendrimers resulting in the release of the encapsulated siRNA cargo was observed between 12–24 h in vitro (Table 2). The formulation transfected human astrocytes after crossing an in vitro BBB model. The transfected siRNA reduced replication of HIV-1 strains X4-HIV NL4-3 and R5-HIV BaL in human astrocyte [159]. Zhou et al. also reported the efficacy of cationic dendrimers as interfering RNA (siRNA) delivery system in humanized mouse model for HIV-1 infection [163] (Table 2). The formulation suppressed HIV-1 infection and provided protection against viral induced CD4(+) T-cell depletion. Follow-up administration of the formulation further resulted in complete inhibition of HIV-1 titers. The formulation accumulate in the peripheral blood mononuclear cells and liver without signs of toxicity indicating that the dendrimers are promising therapeutics for systemic delivery of combinations of siRNAs and the treatment of HIV-1 infection [163]. Briz et al. reported phosphorus-containing dendrimer for the delivery of ODNs and siRNAs. G4 (NH^+^ Et_2_ Cl^−^) 96 formed stable complexes with oligodeoxynucleotides or siRNAs with low cytotoxicity in Sup T1 cells or PBMC. The formulation reduced viral replication significantly indicating that the dendrimers can deliver and transfect siRNA into CD4-T cells as a potential alternative therapy in the HIV-1 infection [171]. Dendrimers are characterized by peripheral active groups and can interact with gp120 or CD4 molecule thereby hindering the attachment of HIV to the host cell. The presence of functional groups also has a huge effect on its antiviral activity. The enhanced cellular uptake of dendrimers also influences its biological activity.

### 5.2. Herpes

**Figure 12:**
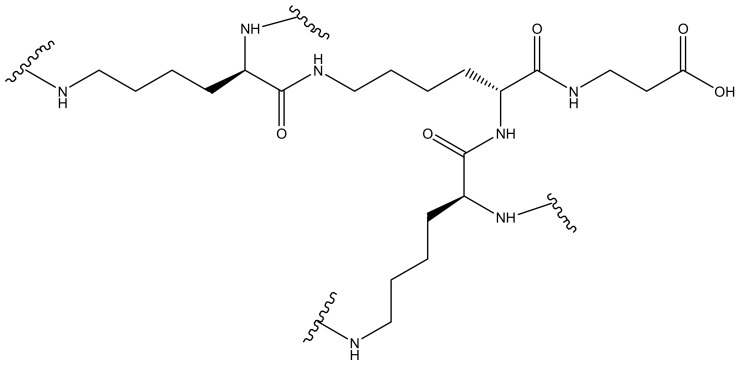
Generic representation of peptide dendrimers.

**Figure 13:**
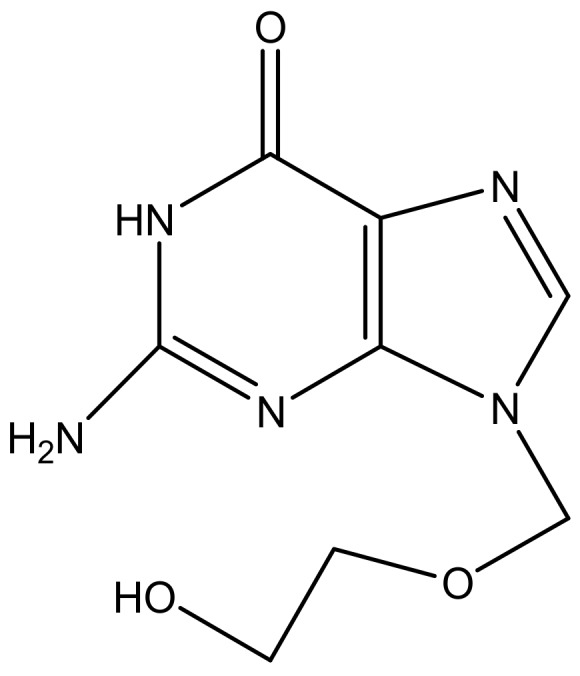
The anti-herpes simplex drug: Acyclovir.

Herpes Simplex Viruses (Type 1 and Type 2) are the most common sexually transmitted infections (STIs) worldwide and they are responsible for a wide variety of clinical infections, including encephalitis, neonatal infections, and or visceral diseases [172]. About 500 million people are currently affected with HSV-2 worldwide and about 20 million new cases occur each year across the world [173]. In South Africa, about 31% of women between the ages 15–26 are infected with HSV-2, and 84% are women who are commercial sex workers in KwaZulu-Natal province [173]. The antivirals that are currently being used have developed resistance, hence there is an increasing need to improve antiviral drugs efficacy [174]. Antiviral drug loaded onto dendrimers have been found to inhibit infections by blocking attachment of the virus to its target cell or tissue [175]. The presence of functional groups on the dendrimers that are able to interact with cell surfaces also result in the killing of the virus [175]. Peptide-derivative dendrimers consist of multiple covalently functional peptides. Peptide dendrimers are synthetic and well-defined macromolecules because they directly inhibit viral infections. They are more effective when combined with other antiviral agents (Figure 12). Lunganini et al. designed peptide-dendrimers and its derivatives (SB105 and SB105-A10) for the inhibition of herpes type 1 and 2. The dendrimers and derivatives were tested for antiviral activity against Vero cells infected with HSV. Both dendrimers derivatives exhibited inhibition HSV adsorption at pH 3.0 and 4.0 and in the presence of 10% human serum proteins, they were also found to prevent type 1 and type 2 herpes virus attachment to the targeted cells. When combined with acyclovir (Figure 13) a high synergistic effect was significant in vitro [150] (Table 2).

Carberry et al. prepared poly (amide)-based dendrimers for viral inhibition. The dendrimers were functionalized with the membrane-peptide gH (625–644) (gH625) derived from the herpes simplex virus type 1 (HSV-1) and encapsulated with glycoprotein H, which is known to be able to deliver cargos into the cellular membrane. The peptide dendrimers showed no sign of cell toxicity with 50% inhibition concentration of 100 nM for HSV-1 and 300 nM for HSV-2 [176]. These results were also similar to the study by Tarallo et al., confirming that peptide- functionalized with gH (625–644) (gH625) dendrimers are promising candidates for intracellular targeted delivery of drugs and the prevention of HSV infection [177]. Ceña-Diez et al. developed polyanionic carbosilane dendrimers with anti-HIV-1 activity as microbicide candidates against sexually transmitted diseases. Plaque reduction assay on Vero cells proved the dendrimers exhibited inhibitory effect against HSV-2 infection. Some of the dendrimers acted by binding directly onto the HSV-2 thereby inactivating while some adhered to host cell-surface proteins. The dendrimers exhibited good synergistic effect with acyclovir and tenofovir against HSV-2, in vitro. Topical vaginal or rectal administration of the formulation prevented HSV-2 transmission in BALB/c mice in values close to 100% [157]. In another research report by Ceña-Diez et al. studied the mechanism of action of peptide derivatized-dendrimers, carbosilane dendrimers, polysulfated galactose functionalized glycodendrimers and PAMAM dendrimers used as microbicides against sexually transmitted diseases (Table 2). These dendrimers were found to act at the stage of viral entry into the target cell by blocking the viral particles that bind to the cell surface heparan sulfate or binding to cellular co-receptors [154]. Tarallo et al. synthesized poly(amide)-based dendrimers functionalized at the termini with a membrane-interacting peptide obtained from herpes simplex virus (HSV) type 1 glycoprotein H, gH625–644 (Table 2). This peptide has been shown to interact with model membranes and to inhibit viral infectivity. The 50% inhibitory concentration of the formulation was 100 and 300 nM against HSV-1 and HSV-2, respectively. These results indicated that functionalization of the dendrimers with the peptide sequence derived from an HSV glycoproteins are promising therapeutics for the treatment of HSV infection [162].

### 5.3. Hepatitis

**Figure 14:**
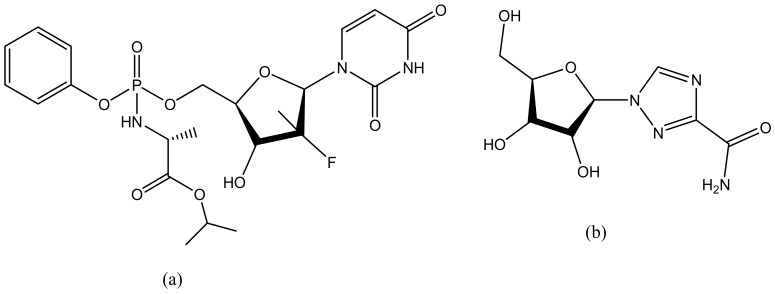
Anti-hepatitis drugs: Sofosbuvir (**a**); Ribavirin (**b**).

Hepatitis is the major cause of chronic liver disease [178]. About 200 million individuals in the world are estimated to suffer from Hepatitis C infection [179]. There is no effective vaccine against hepatitis C and the emergence of transmission of this virus is escalating especially when prophylactic measures are not taken [179]. The antivirals that are currently used such as sofosbuvir (Figure 14a), ribavirin (Figure 14b) have developed resistance to hepatitis C infection, hence there is an urgent need to develop new antiviral agents that will be able to deliver the drugs to its site of action, minimize the side effects, and enhance therapeutic efficacy [180]. Dendrimers are the best candidate for the delivery of antiviral agents. They are biodegradable, biocompatible and can be used as drug carriers [181]. Crespo et al., synthesize polyanionic carbosilane dendrimers for the prevention of hepatitis C virus infection (Table 2). The preliminary studies showed that one of the dendrimers encapsulated with sofosbuvir was able to inhibit the virus infection [156]. Khosravy et al. conjugated hepatitis B virus surface antigen (HBsAg) to dendrimers resulting in the induced high levels of total IgG in vivo [182]. Immunological assays indicated that the immunogenicity of the conjugated HBsAg was enhanced when compared to HBsAg alone [182]. Lakshminarayanan reported liver-targeted dendritic nano-vector functionalized with a galactopyranoside ligand for the delivery of siRNA [161] (Table 2). Targeted delivery of siRNA to the liver was achieved via a highly specific ligand—receptor interaction between dendritic galactose and the asialoglycoprotein receptor. A decrease in HCV RNA levels of 75% was achieved in HCV-JFH1 infectious cell culture systems. The targeted release mechanism of the formulation revealed it is a potential therapeutic for the treatment of infections in the liver such as hepatitis [161].

### 5.4. Influenza

**Figure 15:**
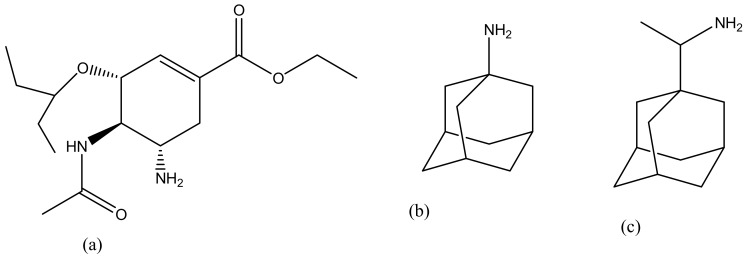
Anti-influenza drugs: Oseltamivir (**a**); Amantadine (**b**); Rimantadine (**c**).

**Figure 16:**
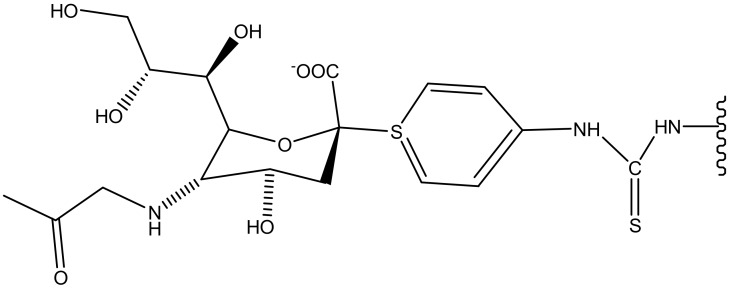
Generation 4 PAMAM sialic acid-based dendrimers.

Influenza is typically treated with antiviral drugs such as oseltamivir (Figure 15a), amantadine (Figure 15b) and rimantadine (Figure 15c) [183,184]. However, oseltamivir has developed some resistance against influenza [185]. The reason of resistance of oseltamivir is that it can lose its ability to bind and inhibit the function of the virus’s NA proteins [186]. Hatano et al. prepared a series of carbosilane dendrimers with hemagglutinin binding peptide against influenza virus (Table 2). The dendrimers showed strong inhibitory activities against human viruses A/PR/8/34 (H1N1) and A/Aichi/2/68 (H3N2) with IC_50_ values of 0.60 µm [155]. Landers et al. conjugated sialic acid-based polyaminoamine dendrimers for the inhibition of hemagglutinin adhesion of three influenza subtype A (H3N2, H2N2 and H3N2). In vivo results showed that the dendrimers were able to permanently inhibit infection caused by H3N2 but were not effective in preventing pneumonitis caused by an H2N2 subtype. Figure 16 shows a schematic representation of generation 4 sialic acid-conjugated polyaminoamine (PAMAM) dendrimers. PAMAM has highly branched functional groups that are useful for the encapsulation of sialic acid. PAMAM exhibited increased delivery of sialic acid and reduced toxicity hence preventing the hemagglutinin adhesion. Dendrimers are promising systems for the delivery of antiviral agents, but issues related to strain specificity must be resolved. Carbosilane dendrimers is the most suitable core scaffold for HA-binding peptide dendrimers [149] (Table 2).

### 5.5. Cervical Cancer

**Figure 17:**
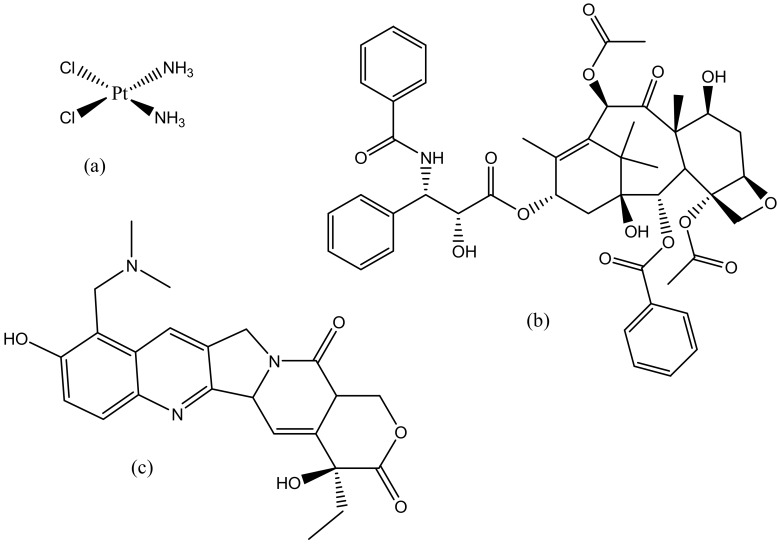
Anticancer drugs: Cisplatin (**a**); Paclitaxel (**b**); Topotecan (**c**).

Cervical cancer is the second most life-threatening cancer among women especially in the developing countries including Africa, Asia and Latin America. Annually more than 200,000 deaths are reported [187]. Cervical cancer can be treated with radiotherapy or surgically if diagnosed at the early stage [188]. Although there are a numerous number of drugs to treat cervical cancer, most of them have developed side effects such as resistance and toxicity [188]. Chemotherapy is one method used to treat cancer; chemotherapy agents used to treat cervix cancer include cisplatin (Figure 17a), paclitaxel (Figure 17b), and topotecan (Figure 17c) [189]. The problem with chemotherapy is that patients often experience side effects such as hair loss, kidney damage and toxicity [190]. Toxicity occurs because the healthy cells are exposed to the toxic effects of the drugs [191]. Dendrimers can overcome poor immunogenicity and reduce the toxicity of peptide-based vaccines against cervical cancer [192]. Human papillomavirus (HPV) is the main cause of cervical cancer; hence there is a need to develop a new therapeutic HPV vaccine. This vaccine must be able to stimulate CD8^+^ cytotoxic T lymphocytes that can eliminate HPV infected cells. E6 and E7 peptide dendrimers have been reported to inhibit the growth of HPV cells. This was confirmed by Lui et al., by developing polymer-peptide dendrimers for the treatment of HPV-related cancers. In vivo results showed that the formulation was able to reduce tumor growth and eliminate E7-expressing TC-1 tumors in mice [193]. Similar findings were also reported by Hussein et al., whereby peptide-dendrimers were found to eliminate over 50% tumor cells in vivo [194].

Mekuria et al. synthesized PAMAM dendrimers conjugated with two targeting moieties, IL-6 and RGB peptide (G4.5-lL6 and G4.5-RGD) for the targeted delivery to Hela cells. Both dendrimers were loaded with doxorubicin with an encapsulation efficiency of 51.3 and 30.1% for G4.5-lL6 and G4.5-RGB, respectively. G4.5-lL5/DOX dendrimers exhibited lower IC_50_, higher drug loading and sustained drug release rate compared to G4.5-RGB/DOX dendrimers [152] (Table 2). It was observed that G4.5-IL6 is a potential carrier for targeted drug delivery of doxorubicin to cervical cancer cells.

Dutta et al. formulated dendrimers-based siRNA against E7 and E6 cervical cancer (Table 2). Formulation of dendrimer-siRNA was done by optimization of nitrogen-to-phosphate targeting green fluorescence. The in vitro results showed that these dendrimers were able to inhibit target genes against E6 and E7 cervical cancer. The formulation was found to exhibit siGFP-entrapment efficiency of 49.76% ± 1.62%, vesicle size of 154 ± 1.73 nm, and zeta potential of +3.21 ± 0.07 mV, and also found to be non-toxic to the cells. These approaches can result in decreased side effects of the drugs used to treat cervical cancer, overcome drug resistance and increase the survival rates of individual infected by cervical cancer [151].

## 6. Conclusions

Dendrimers have been investigated as drug delivery systems for the treatment of viral and parasitic infections. However, there are very few reports on the application of dendrimers for the treatment of parasitic infections. In the treatment of leishmaniasis and toxoplamosis, the potential of the dendrimers to reduce the toxicity associated with amphotericin B and its macrophage targeting ability of the formulation was enhanced resulting in the significant killing of the parasite. The dendrimers were selective by delivering the drug to the parasite reservoir cells, phagocytes. Dendrimers have also been developed as vaccine carriers for the delivery of vaccine for the prevention of schistosomiasis infection which was characterized by IgG2a antibody response with an enhanced production of IL-2 and IFN-γ in vivo. In the treatment of malaria, the dendrimers exhibited specific targeting mechanism to the plasmodium red blood cells when compared to the non-infected red blood cells.

In the treatment of viral infections, dendrimers have the potential to inhibit herpes simplex virus. They hindered HSV-1 and HSV-2 attachment to the target cells. They also blocked the sexual transmission of HIV-1 and destabilized hepatitis C infection. These findings so far suggest that dendrimers are potential delivery systems for treatment of infectious diseases. However, there is a pressing need for more studies in order to fully understand their mode of action.

## Figures and Tables

**Table 1:** Various dendrimers for parasitic diseases.

Dendrimers Classification	Combination with Type of Drugs	Type of Infection	References
PPI	Amphotericin B	Leishmaniasis	[116,118]
PAA	Sulfadoxine, chloroquine and primaquine	Toxoplamosis	[118,119]
PAMAM	DNA Chloroquine and primaquine	Schistosomiasis Malaria	[120] [121]
Poly-l-lysine	Chloroquine	Malaria	[122]

**Table 2:** Various dendrimers for viral diseases.

Dendrimers Classification	Combination with Type of Drugs	Type of Infection	References
PAA	DNA	Influenza	[149]
Peptide dendrimers	Acyclovir	Herpes	[150]
	siRNA	Cervical cancer	[151]
	Doxorubicin	Cervical cancer	[152]
Carbosilane dendrimers	Zidovudine, efarvenz and tenofovir	HIV	[148]
	Maraviroc and tenofovir	HIV	[153]
	Heparan sulfate	Herpes	[154]
	Oseltamivir	Influenza	[155]
	Sofosbuvir	Hepatitis	[156]
	Acyclovir and tenofovir	Herpes	[157]
	Microbicide	HIV	[158]
	siRNA	HIV	[159]
PPI	Zidovudine	HIV	[160]
PETIM	siRNA	Hepatitis	[161]
PA	Glycoprotein H	Herpes	[162]
PAMAM	Heparan sulphate	Herpes	[154]
	siRNA	HIV	[163]
